# Prevalence of Screening for Diabetes Mellitus in Patients Previously Diagnosed with Gestational Diabetes: Factors Related to its Performance

**DOI:** 10.1055/s-0042-1757955

**Published:** 2022-12-29

**Authors:** Lucas Kindermann, Leandro de Liz Costa, Alberto Trapani Júnior

**Affiliations:** 1 Departament of Gynecology and Obstetrics, Hospital Regional de São José Dr. Homero de Miranda Gomes, São José, SC, Brazil; 2 Faculdade de Medicina, Universidade do Sul de Santa Catarina, Palhoça, SC, Brazil

**Keywords:** gestational diabetes, screening programs, postpartum period, diabetes mellitus, diabetes gestacional, programas de rastreamento, período pós-parto, diabetes mellitus

## Abstract

**Objective** To determine how many patients underwent screening for diabetes mellitus (DM) in the puerperium after a diagnosis of gestational DM (GDM) and which factors were related to its performance.

**Methods** The present is a prospective cohort study with 175 women with a diagnosis of GDM. Sociodemographic and clinico-obstetric data were collected through a questionnaire and a screening test for DM was requested six weeks postpartum. After ten weeks, the researchers contacted the patients by telephone with questions about the performance of the screening. The categorical variables were expressed as absolute and relative frequencies. The measure of association was the relative risk with a 95% confidence interval (95%CI), and values of *p* ≤ 0.05 were considered statistically significant and tested through logistic regression.

**Results** The survey was completed by 159 patients, 32 (20.1%) of whom underwent puerperal screening. The mean age of the sample was of 30.7 years, and most patients were white (57.9%), married (56.6%), and had had 8 or more years of schooling (72.3%). About 22.6% of the patients used medications to treat GDM, 30.8% had other comorbidities, and 76.7% attended the postnatal appointment. Attendance at the postpartum appointment, the use of medication, and the presence of comorbidities showed an association with the performance of the oral glucose tolerance test in the puerperium.

**Conclusion** The prevalence of screening for DM six weeks postpartum is low in women previously diagnosed with GDM. Patients who attended the postpartum consultation, used medications to treat GDM, and had comorbidities were the most adherent to the puerperal screening. We need strategies to increase the rate of performance of this exam.

## Resumo

**Objetivo** Determinar quantas pacientes realizaram o rastreamento puerperal para diabetes mellitus (DM) após diagnóstico de DM gestacional (DMG) e quais fatores estão relacionados com a sua realização.

**Métodos** Trata-se de um estudo com uma coorte prospectiva de 175 puérperas com diagnóstico de DMG. Informações sociodemográficas e clinico-obstétricas foram coletadas por meio de questionário, e solicitou-se a realização do rastreamento para DM às seis semanas de pós-parto. Após dez semanas, os pesquisadores contataram as pacientes por telefone com questões sobre a realização do rastreamento. As variáveis categóricas foram expressas em termos de frequências absoluta e relativa. A medida de associação foi o risco relativo com intervalo de confiança de 95% (IC95%), e valores de *p* ≤ 0,05 foram considerados estatisticamente significativos e testados por regressão logística.

**Resultados** A pesquisa foi concluída por 159 pacientes, 32 (20,1%) das quais fizeram o rastreamento puerperal. A idade média da amostra foi de 30,7 anos, e a maioria das pacientes eram brancas (57,9%), casadas (56,6%) e tinham 8 ou mais anos de escolaridade (72,3%). Cerca de 22,6% utilizaram medicações para tratamento da DMG, 30,8% eram portadoras de outras comorbidades, e 76,7% compareceram na consulta pós-natal. O comparecimento na consulta pós-parto, a utilização de medicamentos e a presença de comorbidades demonstraram associação com a realização do teste oral de tolerância à glicose no puerpério.

**Conclusão** O rastreamento de DM após seis semanas de puerpério é baixo em mulheres com diagnóstico prévio de DMG. Pacientes que compareceram na consulta pós-parto, utilizaram medicações para tratamento da DMG, e eram portadoras de comorbidades foram as mais aderentes à realização do rastreamento puerperal. Necessitamos de estratégias para aumentar a abrangência da realização desse exame.

## Introduction

Diabetes mellitus (DM) is a chronic disease characterized by high levels of blood glucose due to insufficient insulin production or some degree of insulin resistance that can contribute to physical, psychological and clinical complications. 1 It is estimated that ∼ 9.3% of the world population of adults aged between 20 and 79 years live with this condition. 1 Brazil is the South-American country with the highest prevalence of this disease, with 16.8 million adults affected, and the prevalence is higher among women (10.4% of the population) than among men (8.4% of the population). 1

Women with hyperglycemia detected for the first time during pregnancy and with blood glucose levels that are not sufficient to achieve the diagnostic criteria for DM are characterized as having gestational DM (GDM). 2 It is estimated that 15.8% of the patients who delivered live newborns worldwide were affected by hyperglycemia in 2019, and 83.6% of the cases were due to GDM. 1

In Brazil, the diagnosis of GDM follows the consensus of the International Association of the Diabetes and Pregnancy Study Group (IADPSG): patients with fasting glucose levels between 92 mg/dL and 125 mg/dL or at least one altered value on the Oral Glucose Tolerance Test (OGTT) with 75 g of dextrose, performed between 24 and 28 weeks, with cutoff points of ≥ 92 mg/dL fasting, ≥ 180 mg/dL in the first hour, and ≥ 153 mg/dL in the second hour. 2 3

When compared with pregnant women with normoglycemia, GDM carriers are ∼ 7 to 10 times more likely to develop type-2 DM (DM2) after delivery. 4 5 The prevalence of hyperglycemia in patients with GDM varies from 25.9% in the first year postpartum to up to 53.7% when evaluated 5 years later. 6 Therefore, national and international protocols recommend performing the OGTT with 75 g of dextrose 6 weeks after delivery to screen for DM in patients diagnosed with GDM. 2 7 Early diagnosis of diabetes is important to avoid the impacts of prolonged hyperglycemia, such as increased incidence and mortality from cardiovascular diseases, since the diagnosis of GDM is also associated with higher rates of dyslipidemia, hypertension, vascular dysfunctions, atherosclerosis, and occurrence of cardiovascular events throughout life. 8 9 10 However, despite the recommendations, the rate of patients diagnosed with GDM who undergo postpartum screening is generally low, ranging from 19.5% to 54%. 11 12

It has been observed that many patients fail to undergo DM screening during the puerperium. There is a need to create a program to follow up these patients, perform exams to enable the establishment of early diagnoses, and act in the prevention and treatment of this comorbidity that has such a great long-term impact for the patient and the public health system. Thus, the present research aimed to determine how many GDM patients among the population under study undergo the screening test in the puerperal period and what are the factors related to its performance.

## Methods

The present is a prospective cohort study. The population consisted of patients diagnosed with GDM, hospitalized for delivery, in the public health system, between May and September 2021, at the Obstetrics service of Hospital Regional de São José Dr. Homero de Miranda Gomes, in the city of São José, state of Santa Catarina, Southern Brazil. The sampling was carried out by convenience, with an estimated sample size of 175 cases. The calculation was performed with the OpenEpi software, version 3, considering the significant variables from previous studies, 13 14 with bilateral significance (1-α) of 0.05 and power (1-β) of 0.80, and an estimated loss of up to 30%.

The inclusion criteria were patients diagnosed with GDM based on the aforementioned IADSPG consensus, 2 3 who knew how to read and write in Portuguese. Patients with blood glucose levels in the laboratory screening that enabled the establishment of a diagnosis of previous DM or who had already had a diagnosis prior to pregnancy were excluded.

At first, during hospitalization, the patients answered a questionnaire through an interview with the researcher pertaining to: sociodemographic data (age, marital status, self-declared skin color, area of residence, years of schooling, family income in terms of the Brazilian minimum monthly wage of $213,09 according to federal law no. 14.158/2021 and commercial US dollar quotation at the beginning of the 2021, occupational status); obstetric history (parity, number of prenatal appointments, pregestational BMI, weight gain during pregnancy, mode of delivery, use of oral antidiabetics or insulin during pregnancy, birth weight, shoulder dystocia, amniotic fluid disorders diagnosed in any moment of pregnancy, gestational age at the time of the GDM diagnosis, history of GDM in previous pregnancies, breastfeeding during hospitalization, and associated comorbidities like hypertensive syndromes, thyroid disorders, and neurological or atopic diseases); family history of DM; and personal habits (sedentary lifestyle, smoking status). The data collected was complemented by the analysis of the hospital records and prenatal care documents. Birth weight greater than 4,000 g, presence of polyhydramnios, occurrence of shoulder dystocia and/or admission to the Neonatal Intensive Care Unit (NICU) were also grouped in a variable named “Composite outcome perinatal morbidity” for the statistical analysis.

The patients were informed of the importance of screening for DM six weeks after delivery. At the time of hospital discharge, they received a request for an OGTT test with 75 g of glucose (before and 2 hours after ingestion), which should be scheduled and performed at the Basic Health Unit of their hometowns, through the Brazilian Unified Health System. 2

After four weeks of the period stipulated for the performance of the screening test, the researchers contacted the patients by telephone, asking if they had performed the requested test and if they kept breastfeeding in this period. The patients who failed to undergo the exam were asked why by the researchers. Patients who presented positive screening were instructed to seek their Basic Health Unit to continue the management of the disease.

After the collection the data was inserted in a Windows Excel (Microsoft Corp., Redmond, WA, United States) spreadsheet and later exported to the Statistical Package for the Social Sciences (PASW Statistics for Windows, SPSS Inc., Chicago, IL, United States) software, version 18.0 for the descriptive and analytical analyses. To describe the quantitative variables, means and standard deviations were calculated. The categorical variables were expressed as absolute (n) and relative (%) frequencies. The bivariate analysis was performed using the Chi-squared test to present the distribution of the variables. The measure of association used was the relative risk (RR) and 95% confidence interval (95%CI). Differences were considered statistically significant when *p* ≤ 0.05, and they were tested through logistic regression.

The present research project was submitted to the Research Ethics Committee and accepted under CAAE number 45699821.1.0000.0110, and data collection only started after the approval.

## Results

**Fig. 1:**
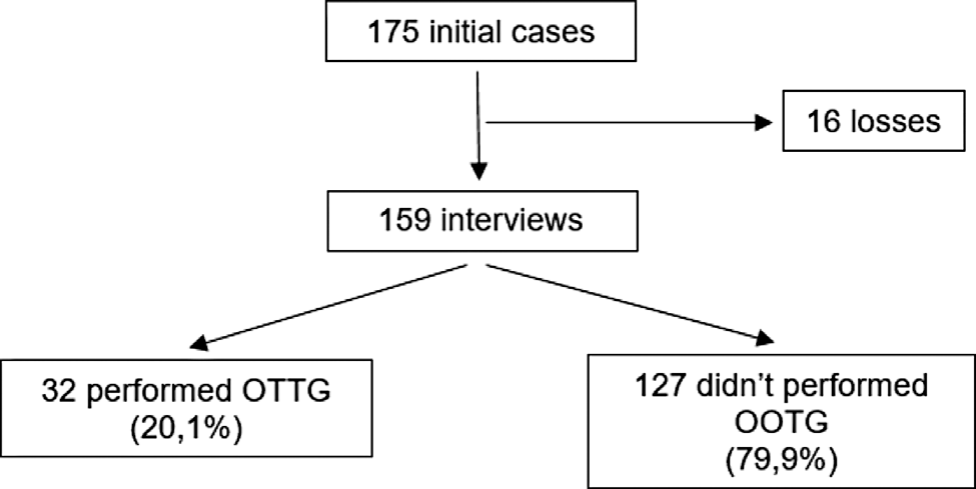
Flowchart of the selection of the 175 patients who compose the study sample.

The sample was composed of 175 puerperal women diagnosed with GDM, and all of them answered the questionnaire. In the second stage, 159 patients completed the survey, with a total of 16 losses: patients who did not answer the telephone contact, or whose number was wrong in the registration, or who requested exclusion from the study during the telephone contact ( Fig. 1 ).

The age of the patients ranged from 17 to 44 years, with a mean of 30.7 years, and most (66.7%) were aged between 20 and 34 years, self-reported as white (57.9%), were married (56.6%), and reported 8 or more years of schooling (72.3%). The other sociodemographic characteristics are shown in Table 1 .

**Table 1:** Sociodemographic characteristics of the 159 patients who completed the survey of the present study

Variables	Underwent the OGTT (%)	Did not undergo the OGTT (%)	Total (%)	*p* -value
*Age*				
Mean	32.5 ± 5.8	30.2 ± 6.1	30.7 ± 5.8	
< 19 years old	1 (11.1)	8 (88.9)	9 (5.7)	0.487
20–34 years old	20 (18.9)	86 (81.1)	106 (66.7)	
> 35 years old	11 (25.0)	33 (75.0)	44 (27.6)	0.343
*Self-declared skin color*				
White	19 (20.7)	73 (79.3)	92 (57.9)	
Non-white (others)	13 (19.4)	54 (80.6)	67 (42.1)	0.846
*Marital status: living with partner*				
Yes	31 (31)	69 (69)	152 (95.6)	0.693
No	1 (14.3)	6 (85.7)	7 (4.4)	
*Years of schooling*				
< 8 years	7 (15.9)	37 (84.1)	44 (27.7)	0.412
≥ 8 years	25 (21.7)	90 (78.3)	115 (72.3)	
*Family income*				
< 1 minimum wage	1 (11.1)	8 (88.9)	9 (5.7)	0.124
Between 1 and 2 minimum wages	6 (13.6)	38 (86.4)	44 (27.7)	
Between 2 and 3 minimum wages	5 (11.6)	38 (88.4)	43 (27.0)	
> 3 minimum wages	20 (31.7)	43 (68.3)	63 (39.6)	
*Pre* gestational BMI				
< 18.5 Kg/m ^2^	0 (00.0)	1 (100.0)	1 (0.6)	
18.5–24.9 Kg/m ^2^	5 (16.1)	26 (83.9)	31 (19.5)	
25–29.9 Kg/m ^2^	15 (26.3)	42 (73.7)	57 (35.9)	
> 30 Kg/m ^2^	12 (17.1)	58 (82.9)	70 (44.0)	0.405
*Occupational status: paid work*				
Yes	19 (24.4)	59 (75.6)	78 (49.0)	0.191
No	13 (16.0)	68 (84.0)	81 (51.0)	
*Family history of DM*				
Yes	18 (22.5)	62 (77.5)	80 (50.3)	0.452
No	14 (17.7)	65 (82.3)	79 (49.7)	
*History of GDM in previous pregnancies*				
Yes	3 (14.3)	18 (85.7)	21 (13.2)	0.474
No	29 (21.0)	109 (79.0)	138 (86.8)	
*Smoking*				
Yes	7 (25.9)	20 (74.1)	27 (17.0)	0.409
No	25 (18.9)	107 (81.1)	132 (83.0)	
*Breastfeeding*				
Yes	27 (20.0)	108 (80.0)	135 (84.9)	0.633
No	5 (20.8)	19 (79.2)	24 (15.1)	

Abbreviations: BMI, body mass index; DM, diabetes mellitus; GDM, gestational diabetes mellitus; OGTT, oral glucose tolerance test.

As for the obstetric data, most patients were not primiparous (73.6%) and had completed prenatal care with more than 6 appointments (83.6%). Of the 159 patients who completed the survey, 76.7% attended the postnatal appointment, 22.6% used medications (oral antidiabetics or insulin therapy) to treat GDM, and 30.8% had other comorbidities. There was 1 case of shoulder dystocia during delivery. The rest of the obstetric data is presented in Table 2 .

**Table 2:** Obstetrics and perinatal characteristics of the 159 patients who completed the survey of the present study

Obstetrics and perinatal characteristics	Underwent the OGTT (%)	Did not undergo the OGTT (%)	Total (%)	*p* -value
*Primiparity*				
Yes	10 (23.8)	32 (76.2)	42 (26.4)	0.488
No	22 (18.8)	95 (81.2)	117 (73.6)	
*Prenatal appointments*				
≤ 6	2 (7.7)	24 (92.3)	26 (16.4)	0.084
> 6	30 (22.6)	103 (77.4)	133 (83.6)	
*Postnatal appointments*				
Yes	30 (24.6)	92 (75.4)	122 (76.7)	0.011
No	2 (5.4)	35 (94.6)	37 (23.3)	
*Use of medications*				
Yes	15 (41.7)	21 (58.3)	36 (22.6)	< 0.001
No	17 (13.8)	106 (86.2)	123 (77.4)	
*Associated comorbidities* *				
Yes	17 (34.7)	32 (65.3)	49 (30.8)	0.002
No	15 (13.6)	95 (86.4)	110 (69.2)	
*Birth weight* > 4,000 g				
Yes	2 (14.3)	12 (85.7)	14 (8.8)	0.568
No	30 (20.7)	115 (79.3)	145 (91.2)	
*Polyhydramnios*				
Yes	1 (20.0)	4 (80.0)	5 (3.1)	0.994
No	31 (20.1)	123 (79.9)	154 (96.9)	
*Mode of delivery*				
Vaginal	16 (22.2)	56 (77.8)	72 (45.3)	
Cesarean section	16 (18.4)	71 (81.6)	87 (54.7)	0.549
*Admission to the Neonatal Intensive Care Unit*				
Yes	9 (27.3)	24 (72.7)	33 (20.8)	0.250
No	23 (18.3)	103 (81.7)	126 (79.2)	
*Composite outcome perinatal morbidity* #				
Yes	8 (17.8)	37 (82.2)	45 (15.7)	0.643
No	24 (21.1)	90 (78.9)	114 (71.7)	

Abbreviations: OGTT, oral glucose tolerance test.

Notes: *Associated comorbidities, such as like hypertensive syndromes, thyroid disorders, and neurological or atopic diseases.

# Birth weight > 4,000 g, polyhydramnios, shoulder dystocia, and admission to the Neonatal Intensive Care Unit.

Of the total of 159 cases, 122 women (76.7%) attended the puerperal appointment. Among those, 43 (35.2%) reported they had been instructed about the OGTT and its performance. In total 32 patients (20.1%) underwent the exam 6 weeks postpartum. Among the 127 women who did not undergo the test, 51 (40.1%) reported having submitted the request for the exam at their Basic Health Unit and that the exam was not scheduled; 32 (25.2%) said they forgot to place the request; 7 (5.5%) reported they were instructed by the Basic Health Unit not to undergo it, or to do so at intervals not stipulated by the present study, and in 37 (29.2%) cases the justifications were different. Among the 32 OGTTs performed in the postpartum period, no case of DM was diagnosed, and glucose intolerance was evidenced in 2 exams. The univariate analysis ( Table 3 ) showed that attending the postnatal consultation, having used medication for GDM, and having comorbidities were factors related to performing the screening test in the puerperium. After logistic regression, these variables remained significant, as shown in Table 4 .

**Table 3:** Analysis of factors associated with the performance of postpartum screening of the 159 patients who completed the survey of the present study

Factors	Univariete analysis		
	Relative risk	95% confidence interval	*p* -value
Postnatal appointment	1.294	1.13–1.49	0.011
Medication use	2.835	1.66–4.85	< 0.001
Associated comorbidities*	2.108	1.35–3.28	0.002
Age < 19 years old	0.487	0.08–3.50	0.487
Age > 35 years old	1.323	0.75–2.32	0.343
Self-declared skin color non-white	0.955	0.60–1.52	0.846
Marital status: living with partner	1.017	0.94–1.09	0.693
< 8 years of schooling	0.751	0.37–1.53	0.412
Family income < 2 minimum wages	0.604	0.30–1.21	0.124
Pregestational BMI > 30 Kg/m ^2^	0.821	0.51–1.33	0.405
Work	1.278	0.91–1.80	0.191
History family of DM	1.152	0.81–1.64	0.452
GDM in previous pregnancies	0.661	0.21–2.11	0.474
Smoking	1.389	0.64–3.00	0.409
Breastfeeding	0.964	0.82–1.13	0.633
Primiparity	1.240	0.68–2.25	0.488
< 6 prenatal appointments	0.331	0.08–1.33	0.084
Birth weight > 4,000 g	0.661	0.16–2.81	0.568
Polyhydramnios	0.992	0.11–8.58	0.994
Cesarean section	0.894	0.61–1.31	0.549
Admission to the NICU	1.488	0.77–2.88	0.250
Composite outcome perinatal morbidity #	0.858	0.44–1.66	0.643

Abbreviations: BMI, body mass index; DM, diabetes mellitus; GDM, gestational diabetes mellitus; NICU, Neonatal Intensive Care Unit.

Notes: *Associated comorbidities such as hypertensive syndromes, thyroid disorders, and neurological or atopic diseases.

# Birth weight > 4,000 g, polyhydramnios, shoulder dystocia, and admission to the Neonatal Intensive Care Unit.

**Table 4:** Logistic regression of the significant factors associated with the performance of postpartum screening of the 159 patients who completed the survey of the present study

Factors		Logistic regression	
	Relative risk	95% confidence interval	*p* -value
Postnatal appointment	1.213	1.11–1.37	0.023
Medication use	2.455	1.54–4.21	< 0.001
Associated comorbidities*	2.017	1.23–3.12	0.004

Note: *Associated comorbidities such as hypertensive syndromes, thyroid disorders, and neurological or atopic diseases.

## Discussion

The focus of the present study is to establish the prevalence of DM screening in the puerperium and what are the factors associated with its performance. In the present study, about 1 in 5 women (20.1%) completed the OGTT screening 6 weeks postpartum. This index is similar to that found by Ortiz et al., 15 who found a rate of 19%. The prevalence of DM screening in patients with a history of GDM is quite variable in the literature, ranging from 13.8% to 41%, but what all the studies 13 14 16 17 18 19 show, including the current study, is that even though screening is indicated for all postpartum women with a history of GDM, its performance is still low. Screening is important, as it enables the establishment of the risks of developing DM, so physicians can prescribe behavioral or even pharmacological changes to prevent or delay the its diagnosis in this population and avoid the impacts of this comorbidity in the long term. 8 9 10 20

The main reason given for not performing the screening was failure to schedule the examination by the Basic Health Unit (40.1%), despite clear guidelines provided by the Brazilian Ministry of Health. 2 This shows the fragility or lack or organizational alignment within the Brazilian public health care network. The other justifications, for the most part, were related to forgetfulness and other personal issues such as early return to work and difficulties with baby care. These cases could be avoided with more comprehensive assistance on the part of health agents.

Women who attended the postpartum appointments were more likely to undergo the screening test (RR: 1.213; 95%CI: 1.11–1.37). Corroborating the present study, Lawrence et al. 21 observed that patients who attended follow-up postnatal appointments were three times more likely to complete the DM screening. This is probably due to the relationship between seeking medical care at the primary care unit with the delivery of the exam request at the Basic Health Unit and the admission into the process of its performance and also the positive feedback on screening guidance during the appointment, although in the present study only 35.2% of the patients who attended the appointment reported having received guidance about the examination by the professionals who assisted them. In another study, 22 whose focus was to observe the relationship between the level of training and the rate of DM screening, the authors found that puerperal women examined by resident physicians or midwives in their postnatal appointment, rather than by fully-trained physicians, tended to adhere more to the performance the test.

Another positive factor for the performance of the OGTT was the association with the need to use medication to treat GDM (RR: 2.455; 95%CI 1.54–4.21). Cabizuca et al. 16 showed that the use of insulin during pregnancy increases screening rates by ∼ 6 times. This may be due to the perception that the use of medication implies a greater severity of the condition, which leads patients to be more concerned about the persistence of the disease in the puerperium, with a consequent greater demand for screening.

In the present study, we were able to show the association between the presence of comorbidities and adherence to screening (RR: 2.017; 95%CI: 1.23- 3.12). To the best of our knowledge, the present is the first study to show the correlation between these factors. This association may be due to the fact that women who have other morbidities, in addition to GDM, are already more concerned about their health; therefore, a higher rate of them chose to undergo screening.

Although these are the only significant associations in the present study, a few other articles 14 17 23 24 25 26 also relate primiparity, advanced maternal age, history of GDM in previous pregnancies and family history of DM as factors that increase patient adherence to screening.

When interpreting the results of the present study, one should take into account that the patients received previous guidance on the performance of the screening and left the hospital with the request for the exam. The results might have been different if the guidance about the importance of the OGTT and the request for it depended on the primary care unit.

## Conclusion

The rate of DM screening through the OGTT six weeks postpartum in women diagnosed with GDM is low. Women who attended the postpartum appointment, who needed medication for the treatment of GDM, and who had comorbidities were the most adherent to screening. It is essential to develop measures to cover a greater number of patients, such as follow-up programs, monitoring to prevent these women from leaving the hospital until the exam is performed, and improvements in the access to information regarding the importance of puerperal screening for DM, mainly in the primary health care setting.
